# Multidrug-Resistant *Escherichia albertii*: Co-occurrence of β-Lactamase and MCR-1 Encoding Genes

**DOI:** 10.3389/fmicb.2018.00258

**Published:** 2018-02-16

**Authors:** Qun Li, Hong Wang, Yanmei Xu, Xiangning Bai, Jianping Wang, Zhengdong Zhang, Xiang Liu, Yimao Miao, Ling Zhang, Xinqiong Li, Nianli Zou, Guodong Yan, Xi Chen, Jie Zhang, Shanshan Fu, Ruyue Fan, Jianguo Xu, Juan Li, Yanwen Xiong

**Affiliations:** ^1^Zigong Center for Disease Control and Prevention, Zigong, China; ^2^Collaborative Innovation Center for Diagnosis and Treatment of Infectious Diseases, State Key Laboratory of Infectious Disease Prevention and Control, National Institute for Communicable Disease Control and Prevention, Chinese Center for Disease Control and Prevention, Beijing, China

**Keywords:** *Escherichia albertii*, β-lactam, β-lactamases, ESBL, *mcr-1*

## Abstract

*Escherichia albertii* is an emerging member of the *Enterobacteriaceae* causing human and animal enteric infections. Antimicrobial resistance among enteropathogens has been reported to be increasing in the past years. The purpose of this study was to investigate antibiotic resistance and resistance genes in *E. albertii* isolated from Zigong city, Sichuan province, China. The susceptibility to 21 antimicrobial agents was determined by Kirby–Bauer disk diffusion method. The highest prevalence was tetracycline resistance with a rate of 62.7%, followed by resistance to nalidixic acid and streptomycin with a rate of 56.9 and 51.0%, respectively. All isolates were sensitive or intermediate susceptible to imipenem, meropenem, amoxicillin–clavulanic acid, and levofloxacin. Among 51 *E. albertii* isolates, 15 were extended-spectrum β-lactamase-producing as confirmed by the double disk test. The main β-lactamase gene groups, i.e., *bla*_TEM_, *bla*_SHV_, and *bla*_CTX-M_, were detected in17, 20, and 22 isolates, respectively. Furthermore, four colistin-resistant isolates with minimum inhibitory concentrations of 8 mg/L were identified. The colistin-resistant isolates all harbored *mcr-1* and *bla*_CTX-M-55_. Genome sequencing showed that *E. albertii* strain SP140150 carried *mcr-1* and *bla*_CTX-M-55_ in two different plasmids. This study provided significant information regarding antibiotic resistance profiles and identified the co-occurrence of β-lactamase and MCR-1 encoding genes in *E. albertii* isolates.

## Introduction

*Escherichia albertii* is a gram-negative facultative rod bacterium belonging to a member of the *Enterobacteriaceae*. It was previously recovered from stool specimens of sick Bangladeshi children and was preliminarily identified as atypical *eae*-positive *Hafnia alvei* (Albert et al., 1991, 1992). In 2003, it was proposed as a new species, named *E. albertii* based on further genotypic and biochemical studies (Huys et al., 2003). *E. albertii* was reported to be the probable cause of death for redpoll finches (*Carduelis flammea*) in Alaska in 2004 (Oaks et al., 2010). In recent years, *E. albertii* was reported to be an emerging human enteropathogen associated with many sporadic infections and outbreaks in humans (Konno et al., 2012; Ooka et al., 2012, 2013; Asoshima et al., 2014; Murakami et al., 2014; Brandal et al., 2015; Inglis et al., 2015). Besides, it has been detected in water and raw meats of animal origin (Felfoldi et al., 2010; Maheux et al., 2014; Asoshima et al., 2015; Lindsey et al., 2015; Maeda et al., 2015; Wang et al., 2016), thus posing a high risk to public health.

Antimicrobials are one of the most successful forms of chemotherapy used in the treatment of infectious diseases by killing or inhabiting the growth of microorganisms. The most important and widely used antimicrobials are β-lactam drugs. In recent years, antimicrobial resistance among Gram-negative bacteria has been reported to be increasing (MacVane, 2017). In *Enterobacteriaceae*, resistance to the β-lactam is mediated by production of β-lactamase enzymes which inactivate the drugs by hydrolyzing the β-lactam ring (Bush and Bradford, 2016). Some clinically most important enzymes are as follows: (1) extended-spectrum β-lactamases (ESBLs), including SHV, TEM and CTX-M types; (2) carbapenemases, including class A (KPC types), class B metallo-β-lactamases (MBLs), and class D oxacillinases; and (3) the AmpC cephalosporinases (Bonomo, 2017).

Colistin is an antibiotic of last resort for the treatment of extensively drug-resistant Gram-negative bacteria (Kaye et al., 2016). In a recent study, the emergence of plasmid-mediated colistin resistance has been reported in *E. coli*, raising a great concern around the world (Liu et al., 2016). Thereafter, plasmid-mediated colistin resistance gene (*mcr-1*) has also been identified in other members of the *Enterobacteriaceae* from South America, Asia, Europe and Africa, suggesting that *mcr-1* might be widespread (Olaitan et al., 2016; Al-Tawfiq et al., 2017).

To date, little is known about the antibiotic resistance of *E. albertii*. This study investigated the antibiotic resistance, identified the ESBL-producing and colistin-resistant isolates, and determined the distribution of β-lactamase genes and *mcr-1* gene in *E. albertii* isolated from Zigong city, Sichuan province, China.

## Materials and Methods

### Bacterial Isolates

A total of 51 isolates of *E. albertii* were recovered from various samples collected in Zigong city, Sichuan province between 2013 and 2015, including diarrheal patient feces (3), healthy carrier feces (3), duck intestine (19), chicken intestine (18), chicken meat (3), duck meat (2), raw mutton (1), raw pork (1), and egret excrement (1). All isolates were confirmed to be *E. albertii* based on combination of diagnostic multiplex PCR, 16S rDNA sequencing, and multi-locus sequence typing (MLST) analysis as described previously (Wang et al., 2016) and stored at -80°C in Luria-Bertani (LB) medium (Oxoid, United Kingdom) with 30% (vol/vol) glycerol.

### Antibiotic Susceptibility Testing

Susceptibility to antimicrobials was determined by Kirby–Bauer disk diffusion method on Mueller Hinton agar (MHA). The antibiotics used in the study included imipenem (IMP), meropenem (MEM), piperacillin (PRL), ampicillin/sulbactam (SAM), amoxicillin/clavulanic acid (AMC), cefepime (FEP), cefuroxime (CXM), cephalothin (KF), ceftriaxone (CRO), aztreonam (ATM), kanamycin (K), streptomycin (S), gentamicin (CN), nalidixic acid (NA), levofloxacin (LEV), norfloxacin (NOR), ciprofloxacin (CIP), trimethoprim/sulfamethoxazole (SXT), tetracycline (TE), furadantin (F), and chloramphenicol (C) (Oxoid, United Kingdom). The inoculated plates were incubated for 24 h aerobically at 37°C. The diameters of the zones of inhibition was interpreted according to the Clinical Laboratory Standards Institute (CLSI) guidelines (CLSI, 2016).

The minimum inhibitory concentration (MIC) of colistin was determined by broth microdilution method recommended by the joint CLSI-EUCAST Polymyxin Breakpoints Working Group^1^.

### Identification of the ESBL-Producing Isolates

The double disk test was performed to confirm the ESBL phenotype. It was carried out on MHA with two pairs of disks (ceftazidime + ceftazidime/clavulanic acid and cefotaxime + cefotaxime/clavulanic acid) (BD Diagnostics, United States). The results were interpreted as recommended by the CLSI (CLSI, 2016). *E. coli* ATCC 25922 and *Klebsiella pneumoniae* ATCC 700603 were used as the quality control strains.

### Detection of the β-Lactamase Gene Groups

*Escherichia albertii* isolates were inoculated on LB agar and incubated overnight at 37°C. A colony was suspended in 50 μl of sterilized distilled water and boiled for 10 min. The cell suspension was centrifuged at 10,000 × *g* for 5 min, and the supernatant was used as template DNA. PCR was performed to screen the main β-lactamase gene groups, i.e., *bla*_TEM_, *bla*_SHV_, *bla*_CTX-M_, *bla*_KPC_, and *bla*_NDM_ genes. All primers and PCR conditions used in this study were presented in **Supplementary Table S1**. Each reaction tube contained 10 μl of master Mix (Qiagen, Germany), 0.5 μM of forward and reverse primers, and 1 μl of template DNA, and was made up to a total volume of 20 μl with sterile distilled water.

### Detection and Sequencing of the *mcr-1* Gene

All 51 isolates were subjected to PCR for the presence of *mcr-1* gene using primers described previously (**Supplementary Table S1**). The expected PCR products were sequenced using the ABI 3730 Automated DNA Analyzer (Applied Biosystems, United States).

The *mcr-1* sequences obtained in this study have been deposited in GenBank under accession numbers: KX765477–KX765480.

### Plasmid Profiling

Plasmid DNA profiles of all 51 *E. albertii* isolates were analyzed using the S1-nuclease pulsed-field gel electrophoresis (PFGE) method. Briefly, the bacterial cells embedded in agarose were lysed by SDS/proteinase K, and then were digested with 8 U S1-nuclease at 37°C for 30 min. Finally, each sample was resolved by PFGE in a Chef-Mapper (Bio-Rad, United States) at 14°C, with a switch time 2.16 to 54.17 s at 6 V/cm for 18 h. Each DNA band visualized was considered as a unit length of linear plasmid. The approximate size of each plasmid was determined by comparing profiles with *Xba*I-digested DNA from *Salmonella* serotype Braenderup strain H9812 (Bai et al., 2017).

### Mating Experiments

Four of the *mcr-1*-positive isolates were selected for conjugation experiments. Filter conjugation was carried out using *E. coli* J53 (sodium azide-resistant) as the recipient. The donor and recipient were grown on LB medium to an optical density at 600 nm of 0.5, mixed equally, and then incubated on sterilized filter paper for 4 h. The filter was then resuspended in LB medium, and dilutions were plated on M-H agar containing sodium azide (150 μg/mL and colistin (4 μg/mL) to select for transconjugants. Mobilization efficiency was calculated as the number of transconjugant colonies divided by the number of donor colonies (Wang et al., 2011).

### Whole-Genome Sequencing

Genomic DNA was isolated from an overnight culture using the Wizard Genomic DNA purification kit (Promega, United States) according to the manufacturer’s instructions. Total DNA obtained was subjected to quality control by agarose gel electrophoresis and quantified by Qubit (Life Technologies, United States). The complete genome was sequenced by single molecule real-time (SMRT) technology using the Pacific Biosciences (PacBio) sequencing platform performed at the Beijing Novogene Bioinformatics Technology, Co., Ltd. (McCarthy, 2010). The filtered reads were assembled to generate one contig without gaps using SMRT Analysis 2.3.0 (Berlin et al., 2015). The protein-coding sequences (CDSs) were predicted using GeneMarkS (Besemer et al., 2001). ARDB (Antibiotic Resistance Genes Database)^2^ was used to search for antimicrobial resistance genes (Liu and Pop, 2009).

The complete genome sequences of SP140150 isolate are available at GenBank under the accession numbers: CP025676–CP025679.

### Ethics Statement

Samples were collected and detected as part of the infectious disease surveillance program led by National Institute for Communicable Disease Control and Prevention, China CDC and implemented by Zigong Center for Disease Control and Prevention. The study was approved by the ethics committee of National Institute for Communicable Disease Control and Prevention, China CDC, according to the medical research regulations of National Health and Family Planning Commission of the People’s Republic of China.

## Results

### Antibiotic Resistance of *E. albertii* Isolates

**FIGURE 1:**
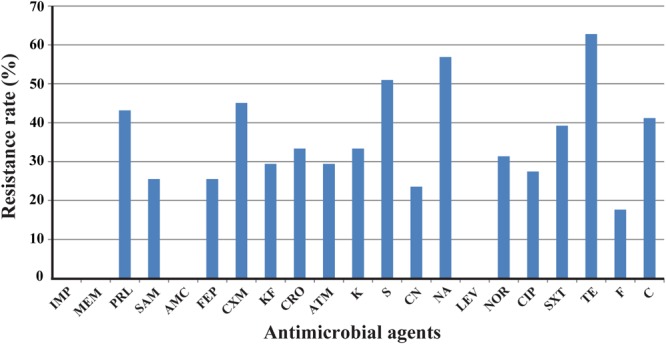
Frequency of antimicrobial resistance of 51 *Escherichia albertii* isolates. IMP, imipenem; MEM, meropenem; PRL, piperacillin; SAM, ampicillin/sulbactam; AMC, amoxicillin/clavulanic acid; FEP, cefepime; CXM, cefuroxime; KF, cephalothin; CRO, ceftriaxone; ATM, aztreonam; K, kanamycin; S, streptomycin; CN, gentamicin; NA, nalidixic acid; LEV, levofloxacin; NOR, norfloxacin; CIP, ciprofloxacin; SXT, trimethoprim/sulfamethoxazole; TE, tetracycline; F, furadantin; C, chloramphenicol.

Antimicrobial resistance in *E. albertii* isolates was determined against 21 antibiotics. The highest prevalence was tetracycline resistance with a rate of 62.7% (32/51), followed by resistance to nalidixic acid and streptomycin with a rate of 56.9% (29/51) and 51.0% (26/51), respectively. Resistance rate to cefuroxime, piperacillin, and chloramphenicol was 45.1, 43.1, and 41.2%, respectively. Lower resistance was observed for ampicillin/sulbactam, cefepime, cephalothin, ceftriaxone, aztreonam, kanamycin, gentamicin, norfloxacin, ciprofloxacin, trimethoprim/sulfamethoxazole, and furadantin with a rate ranging from 17.6 to 39.2%. All isolates were sensitive or intermediate susceptible to imipenem, meropenem, amoxicillin–clavulanic acid, and levofloxacin (**Figure 1**).

Ten isolates (19.6%) were resistant to one antimicrobial agent, while the majority exhibited resistance to two or more antimicrobials tested. All isolates from diarrheal patients and healthy carriers were susceptible to imipenem, meropenem, piperacillin, cefepime, aztreonam, and levofloxacin (**Supplementary Table S2**).

### ESBL-Producing *E. albertii* Isolates

Among 51 *E. albertii* isolates, 15 isolates from four sources, i.e., six isolates from duck intestine, six from chicken intestine, two from chicken meat and one from raw mutton samples, were ESBL-producing as confirmed by the double disk test. All isolates recovered from diarrheal patients and healthy carriers were non-ESBL-producing (**Supplementary Table S2**).

All ESBL-producing *E. albertii* isolates were resistant to piperacillin, cefuroxime, cephalothin, ceftriaxone, aztreonam, tetracycline and chloramphenicol, whereas resistance to streptomycin and nalidixic acid were observed in 14 of the 15 ESBL-producing isolates. ESBL-producing *E. albertii* isolates have higher resistance rate than non-ESBL-producing isolates, as expected (**Table 1**).

**Table 1:** Comparison of the resistance rates of ESBL-producing and of non-ESBL-producing *E. albertii* isolates.

Antibiotic	Non-ESBL-producing (*N* = 36) Resistant [n (%)]	ESBL-producing (*N* = 15) Resistant [n (%)]
Imipenem	0 (0)	0 (0)
Meropenem	0 (0)	0 (0)
Piperacillin	7 (19.4)	15 (100)
Ampicillin/sulbactam	6 (16.7)	7 (46.7)
Amoxycillin/clavulanic acid	0 (0)	0 (0)
Cefepime	0 (0)	13 (86.7)
Cefuroxime	8 (22.3)	15 (100)
Cephalothin	0 (0)	15 (100)
Ceftriaxone	2 (5.6)	15 (100)
Aztreonam	0 (0)	15 (100)
Kanamycin	6 (16.7)	11 (73.3)
Streptomycin	12 (33.3)	14 (93.3)
Gentamicin	7 (19.4)	5 (33.3)
Nalidixic acid	15 (41.7)	14 (93.3)
Levofloxacin	0 (0)	0 (0)
Norfloxacin	6 (16.7)	10 (66.7)
Ciprofloxacin	4 (11.1)	11 (73.3)
Sulfamethoxazole	12 (33.3)	8 (53.3)
Tetracycline	17 (47.2)	15 (100)
Furadantin	0 (0)	9 (60.0)
Chloramphenicol	6 (16.7)	15 (100)

### Distribution of β-Lactamase Genes

The main β-lactamase gene groups (*bla*_TEM_, *bla*_SHV_, *bla*_CTX-M_, *bla*_KPC_, and *bla*_NDM_) were screened by PCR. Eight *E. albertii* isolates did not contained any β-lactamase from gene groups tested and none was positive for *bla*_KPC_ and *bla*_NDM_. The *bla*_TEM_, *bla*_SHV_, and *bla*_CTX-M_ were detected in 17 (33.3%), 20 (39.2%), and 22 (43.1%) isolates, respectively. All 15 ESBL-producing isolates contained 1–3 β-lactamase genes (**Supplementary Table S2**).

### *mcr-1*-Positive and Colistin-Resistant *E. albertii* Isolates

Four out of 51 isolates (one from raw mutton, one from raw chicken meat, and two from chicken intestine) were positive for MCR-1 encoding gene by PCR. Sequencing analysis showed that the four nucleotide sequences are identical to the first reported *mcr-1* sequence in plasmid pHNSHP45 (GenBank accession number KP347127).

The MICs of colistin of all 51 *E. albertii* isolates were determined by broth microdilution method. The four *mcr-1*-positive isolates were colistin-resistant with MIC of 8 mg/L. The MICs of colistin of all *mcr-1*-negative isolates were less than 4 mg/L.

The colistin resistance genes of all four *mcr-1*-positive isolates were successfully transferred by conjugation into sodium azide resistant *E. coli* J53. The transfer frequencies of isolates SP140128, SP140089, SP140149, and SP140150 were similar (4.2 × 10^-3^, 8.8 × 10^-4^, 3.8 × 10^-4^, and 1.3 × 10^-3^, respectively).

### Co-occurrence of *bla*_CTX-M_ and *mcr-1* Genes in *E. albertii* Isolates

Except one isolate T150248 from healthy carrier, plasmids ranging in size from 36 to 283 kbp were identified by S1-nuclease-based PFGE in 50 *E. albertii* isolates. Among which, 23 isolates harbored one plasmid; 13 harbored two plasmids; 10 harbored three plasmids; three harbored four plasmids; and one isolate harbored five plasmids (**Supplementary Table S2**). Two different size plasmids (56 and 113 kbp) were identified in all four colistin-resistant isolates, and an additional 45 kbp plasmid was present in three out of four colistin-resistant isolates (**Table 2**).

**Table 2:** Antimicrobial resistance profiles, β-lactamase genes, and plasmid content of colistin-resistant *E. albertii* isolates.

Isolate	Origin	Antibiotic resistance profile	β-Lactamase gene^∗∗^	Plasmids (kb)^∗∗∗^
SP140128	Mutton	PRL, SAM^∗^, FEP, CXM, KF, CRO, ATM, K, S, NA, NOR, CIP, SXT^∗^, TE, F^∗^, C	*bla*_CTX-M-55_	113, 56
SP140089	Chicken meat	PRL, SAM^∗^, FEP, CXM, KF, CRO, ATM, K, S, NA, NOR, CIP^∗^, SXT^∗^, TE, F, C	*bla*_CTX-M-55_	113, 56, 45
SP140149	Chicken intestine	PRL, SAM^∗^, FEP, CXM, KF, CRO, ATM, K, S, NA, LEV^∗^, NOR, CIP, SXT^∗^, TE, F^∗^, C	*bla*_CTX-M-55_	113, 56, 45
SP140150	Chicken intestine	PRL, SAM^∗^, FEP, CXM, KF, CRO, ATM, K, S, NA, NOR, CIP, SXT^∗^, TE, F, C	*bla*_CTX-M-55_	113, 56, 45

*PRL, piperacillin; SAM, ampicillin/sulbactam; FEP, cefepime; CXM, cefuroxime; KF, cephalothin; CRO, ceftriaxone; ATM, aztreonam; K, kanamycin; S, streptomycin; NA, nalidixic acid; LEV, levofloxacin; NOR, norfloxacin; CIP, ciprofloxacin; SXT, trimethoprim/sulfamethoxazole; TE, tetracycline; F, furadantin; C, chloramphenicol. ^∗^Intermediate sensitivity to antibiotics. ^∗∗^*bla*_CTX-M-55_ type was determined by sequencing. ^∗∗∗^Plasmid sizes were estimated by S1-nuclease PFGE method.*

All four isolates harboring *mcr-1* were positive for *bla*_CTX-M_ group gene (*bla*_CTX-M-55_ type) and were ESBL-producing. The four colistin-resistant isolates showed indistinguishable PFGE patterns and they were all typed as sequence type (ST) 4479 (**Supplementary Figure S1**). They exhibited multi-drug resistance. All four isolates were resistant to 12 antibiotics tested in this study, i.e., piperacillin, cefepime, cefuroxime, cephalothin, ceftriaxone, aztreonam, kanamycin, streptomycin, nalidixic acid, norfloxacin, tetracycline, and chloramphenicol (**Table 2**).

### Genome Features of *E. albertii* Strain SP140150 Harboring *mcr-1*

**FIGURE 2:**
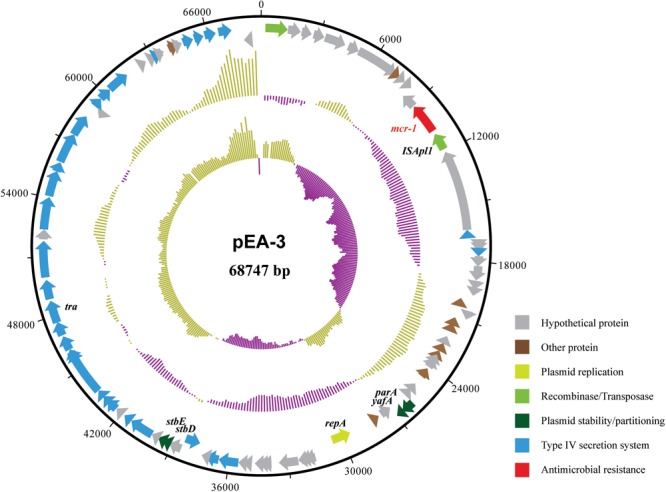
Structure of plasmid pEA-3 carrying *mcr*-1 from *E. albertii* strain SP140150. From outer circle to inner circle, each represents CDS, GC content and GC skew, respectively. The functions of corresponding CDSs are colored as indicated.

The completed genome sequence of SP140150 consists of a circular chromosome of 4,881,553 bp with an average GC content of 49.8% and three circular plasmids. Antimicrobial resistance genes were searched against these three plasmids. *bla*_CTX-M-55_ was identified in pEA-1, a plasmid of 129,356 bp in size with an average GC content of 51.9%. In addition, pEA-1 also carried chloramphenicol resistance gene *cml*, tetracycline resistance determinant *tetA*, streptomycin resistance genes *aph6id* and *aph33ib*, sulfonamide-resistant dihydropteroate synthase gene *sul*2, and multidrug efflux RND transporter OqxA and OqxB. None of any antimicrobial resistance genes was identified in pEA-2, the second plasmid of 57,110 bp in size with an average GC content of 46.1%. A 1626 bp CDS encoding MCR-1 was found located downstream of an insertion sequence *ISApl1* in the third plasmid pEA-3. pEA-3 is 68,747 bp in size with an average GC content of 42.5%. pEA-3 possesses an IncFII-type backbone and contains 90 predicted CDSs encoding plasmid replication, maintenance and stability functions, and a type IV protein secretion system (**Figure 2**).

## Discussion

Antimicrobial resistance is an increasingly encountered phenomenon among the *Enterobacteriaceae* and an alarming threat to global health (Laxminarayan et al., 2013). Resistance to β-lactams is primarily because of bacterially produced β-lactamases that are able to hydrolyze the β-lactam ring (Bush and Bradford, 2016). There are several reports of porin-mediated resistance in clinical isolates of enterobacteria, mainly affecting susceptibility to β-lactams (Martinez-Martinez et al., 1996; Poirel et al., 2004; Pavez et al., 2008; Perez et al., 2013).

Some *E. albertii* isolates in this study demonstrated resistance to four antimicrobials identified by the World Health Organization (WHO) as being of critical importance in the treatment of human infectious diseases, including piperacillin, ampicillin, cefotaxime, and cefepime (WHO, 2012). Furthermore, 29.4% of isolates demonstrated resistance to cephalothin that was identified by the WHO as highly important in human disease treatment (WHO, 2012).

Extended-spectrum β-lactamases are defined as enzymes produced by certain bacteria that are able to hydrolyze extended spectrum cephalosporin. TEM-_group_ (exception of TEM-1 and TEM-2), SHV-_group_, and CTX-M-_group_ β-lactamases are important types of ESBLs (Ghafourian et al., 2015). Among the three types, the prevalence of CTX-M is increasing in *Enterobacteriaceae* and predominates as a cause of extended spectrum cephalosporin resistance (Falagas and Karageorgopoulos, 2009). In the current study, the prevalence of *bla*_CTX-M_ gene in *E. albertii* was in agreement with those reported in the other studies (Bora et al., 2014). Besides, *bla*_CTX-M_ gene was mostly detected in isolates resistant to cefotaxime. Nineteen (37.3%) of *E. albertii* isolates carried two or three different β-lactamase gene groups, demonstrating the co-occurrence of these genes in various combinations (Shahid et al., 2011).

Carbapenem-resistant *Enterobacteriaceae* have been increasingly reported worldwide. The carbapenemases include NDMs, KPCs, OXA-48, and others (Tzouvelekis et al., 2012). KPCs are currently the most common cause of carbapenem resistance worldwide. The emergence of New Delhi metallo-β-lactamase (NDM-1) and its variants had raised a major public health concern. NDM-1 can hydrolyze a wide range of β-lactam antibiotics, including carbapenems (Khan et al., 2017). In China, Wang et al. (2015) has reported that *Enterobacteriaceae* remained susceptible to carbapenems. In the present study, none of the isolate was positive for *bla*_KPC_ or *bla*_NDM_ and all isolates demonstrated susceptibility to meropenem and imipenem.

Polymyxins are active against most members of the *Enterobacteriaceae* family, however, some are naturally resistant to polymyxins, like *Proteus, Brucella, Legionella, Campylobacter*, and *Vibrio* (Poirel et al., 2017). In additional to intrinsic resistance, mechanisms responsible for acquired resistance to polymyxins in *Enterobacteriaceae* have been identified, including genes encoding LPS-modifying enzymes; regulators of the PmrAB and PhoPQ two-component systems; the intrinsic regulator RamA (Poirel et al., 2017). Recently, Liu et al. (2016) had reported plasmid-mediated colistin resistance in *E. coli*. Thereafter, plasmid-mediated colistin resistance gene (*mcr-1*) has been proved to be widespread in other members of the *Enterobacteriaceae* (Olaitan et al., 2016). In this study, four genetically related *E. albertii* isolates were positive for *mcr-1* and were colistin-resistant. When the *mcr-1* harboring plasmid pEA-3 sequence was compared using BLASTn to the nucleotide database at NCBI (accessed 26.01.2018), several highly similar (99% identities with query coverage over 90%) *mcr-1* harboring plasmids from *E. coli, Cronobacter sakazakii* or *Salmonella enterica* were identified, suggesting that *mcr-1* has also spread to *E. albertii*.

## Conclusion

The present study provides significant information regarding antibiotic resistance of *E. albertii* from human, animal, and raw retail meats for the first time. Co-occurrence of β-lactamase and MCR-1 encoding genes in *E. albertii* isolates were identified. Further epidemiological assessments on the drug resistance patterns of *E. albertii* and determination of the molecular resistance mechanisms are needed in the treatment and prevention of both human and animal infections.

## Author Contributions

QL, HW, YM, JX, and YX designed the project; QL, HW, ZZ, XLiu, LZ, GY, XC, and JZ carried out the sampling work; YXu, XB, JW, XLi, NZ, SF, RF, and JL carried out the experiments and generated the data; XLi, JL, and YX analyzed the data and drafted the manuscript.

## Conflict of Interest Statement

The authors declare that the research was conducted in the absence of any commercial or financial relationships that could be construed as a potential conflict of interest.

## Footnotes

1 http://www.eucast.org/fileadmin/src/media/PDFs/EUCAST_files/General_documents/Recommendations_for_MIC_determination_of_colistin_March_2016.pdf

2 http://ardb.cbcb.umd.edu/

## Supplementary Material

FIGURE S1 Pulsed-field gel electrophoresis (PFGE) profiles of four *mcr-1*-positive *E. albertii* isolates. Genomes of *E. albertii* isolates were digested with *Xba* I and fragments separated on a 1% agarose gel using a CHEF-DR III PFGE apparatus, according to the protocol for *E. coli* O157:H7 from PulseNet, United States (http://www.cdc.gov/pulsenet/pathogens/index.html). Multi-locus sequence typing (MLST) was done according to the *E. coli* MLST website (http://mlst.warwick.ac.uk/mlst/dbs/Ecoli).

TABLE S1 PCR primers used to screen the β-lactamase and MCR-1 encoding genes.

TABLE S2 Antibiotic resistance patterns and prevalence of β-lactamase and MCR-1 encoding genes in *E. albertii* isolates.
