# Supplementary figures and images for: Multidrug-Resistant Escherichia albertii: Co-occurrence of β-Lactamase and MCR-1 Encoding Genes
Source: Front Microbiol. 2018 Feb 16;9:258. doi: 10.3389/fmicb.2018.00258 (PMC5820351; doi:10.3389/fmicb.2018.00258)

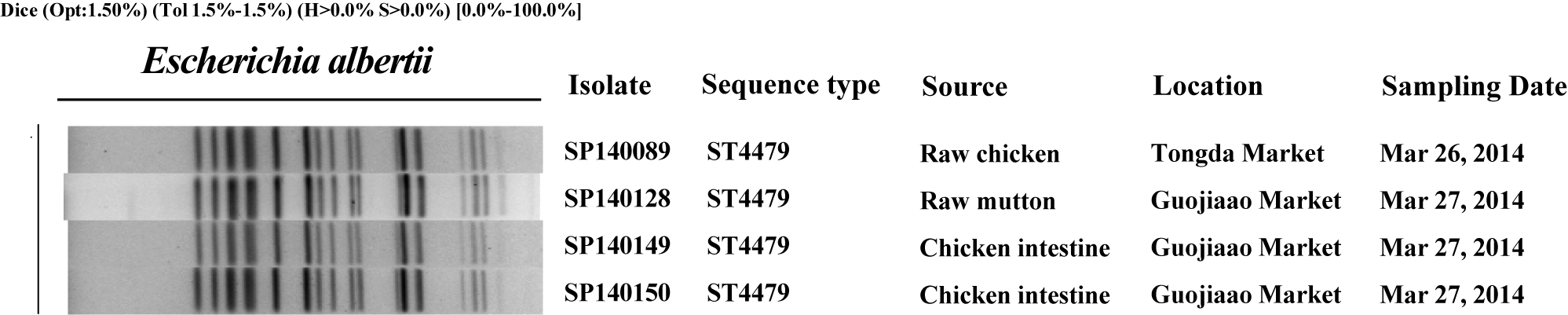

Supplement: FIGURE S1 — Pulsed-field gel electrophoresis (PFGE) profiles of four mcr-1-positive E. albertii isolates. Genomes of E. albertii isolates were digested with Xba I and fragments separated on a 1% agarose gel using a CHEF-DR III PFGE apparatus, according to the protocol for E. coli O157:H7 from PulseNet, United States (http://www.cdc.gov/pulsenet/pathogens/index.html). Multi-locus sequence typing (MLST) was done according to the E. coli MLST website (http://mlst.warwick.ac.uk/mlst/dbs/Ecoli). [file Image_1.TIF]
