# Supplementary material for: Multidrug-Resistant Escherichia albertii: Co-occurrence of β-Lactamase and MCR-1 Encoding Genes
Source: Front Microbiol. 2018 Feb 16;9:258. doi: 10.3389/fmicb.2018.00258 (PMC5820351; doi:10.3389/fmicb.2018.00258)
Supplement: TABLE S1 — PCR primers used to screen the β-lactamase and MCR-1 encoding genes. [file Table_1.DOCX]

**TABLE S1 | PCR primers used to screen the β-lactamase and MCR-1 encoding genes**

| **Target genes** | **Oligonucleotide sequence (5’-3’)** | **Annealing temperature (°C)** | **Amplicon size (bp)** | **References** |
| --- | --- | --- | --- | --- |
| *bla*_SHV_ | F: GAGCGAAAGATCCACTATCG | 55 | 525 | (Haeggman et al., 2004) |
|  | R: GGTATCCCGCAGATAAATCA |  |  |  |
| *bla*_TEM_ | F: ATGAGTATTCAACATTTCCGTG | 54 | 847 | (Zou et al., 2011) |
|  | R: TTACCAATGCTTAATCAGTGAG |  |  |  |
| *bla*_CTX-M_ | F: CAGGAGTTTGAGATGATGAG | 55 | 910 | (Wang et al., 2012) |
|  | R: GAGCGCTCCACATTTTTTAG |  |  |  |
| *bla*_KPC_ | F: GCTACACCTAGCTCCACCTTC | 55 | 945 | (Zou et al., 2011) |
|  | R: TGGAGGGCCAATAGATGATT |  |  |  |
| *bla*_NDM_ | F: CAGGCAACAGCCGAACGA | 54 | 1193 | (Li et al., 2014) |
|  | R: CGTTAGATTGGCTTACACCATTAGA |  |  |  |
| *mcr-1* | F: ATGATGCAGCATACTTCTGTG | 55 | 1626 | (Ye et al., 2016) |
|  | R: TCAGCGGATGAATGCGGTG |  |  |  |

**Reference**

Haeggman, S., Lofdahl, S., Paauw, A., Verhoef, J., and Brisse, S. (2004). Diversity and evolution of the class A chromosomal beta-lactamase gene in *Klebsiella pneumoniae*. *Antimicrob Agents Chemother* 48**,** 2400-2408.

Li, J., Lan, R., Xiong, Y., Ye, C., Yuan, M., Liu, X., Chen, X., Yu, D., Liu, B., Lin, W., Bai, X., Wang, Y., Sun, Q., Wang, Y., Zhao, H., Meng, Q., Chen, Q., Zhao, A., and Xu, J. (2014). Sequential isolation in a patient of *Raoultella planticola* and *Escherichia coli* bearing a novel ISCR1 element carrying blaNDM-1. *PLoS One* 9**,** e89893.

Wang, X.R., Chen, J.C., Kang, Y., Jiang, N., An, S.C., and Gao, Z.C. (2012). Prevalence and characterization of plasmid-mediated blaESBL with their genetic environment in *Escherichia coli* and *Klebsiella pneumoniae* in patients with pneumonia. *Chin Med J (Engl)* 125**,** 894-900.

Ye, H., Li, Y., Li, Z., Gao, R., Zhang, H., Wen, R., Gao, G.F., Hu, Q., and Feng, Y. (2016). Diversified *mcr-1*-Harbouring Plasmid Reservoirs Confer Resistance to Colistin in Human Gut Microbiota. *MBio* 7**,** e00177.

Zou, L.K., Wang, H.N., Zeng, B., Zhang, A.Y., Li, J.N., Li, X.T., Tian, G.B., Wei, K., Zhou, Y.S., Xu, C.W., and Yang, Z.R. (2011). Phenotypic and genotypic characterization of beta-lactam resistance in *Klebsiella pneumoniae* isolated from swine. *Vet Microbiol* 149**,** 139-146.
