# Supplementary material for: Multidrug-Resistant Escherichia albertii: Co-occurrence of β-Lactamase and MCR-1 Encoding Genes
Source: Front Microbiol. 2018 Feb 16;9:258. doi: 10.3389/fmicb.2018.00258 (PMC5820351; doi:10.3389/fmicb.2018.00258)
Supplement: TABLE S2 — Antibiotic resistance patterns and prevalence of β-lactamase and MCR-1 encoding genes in E. albertii isolates. [file Table_2.doc]

**TABLE S2 | Antibiotic resistance patterns and prevalence of β-lactamase and MCR-1 encoding genes in *E. albertii* isolates**

| **Isolate ^a^** | **Origin** | **Antibiotic resistance profile** | **Presence of antibiotic genes** | **Plasmid(s)(kbp)** |
| --- | --- | --- | --- | --- |
| ZG141049 | Diarrheal patient | CRO, TE, CXM*, S* | — | 127 |
| ZG140067 | Diarrheal patient | NA, CXM* | — | 79, 38 |
| D140513 | Egret excrement | CXM* | *bla*_SHV_*, bla*_CTX-M_ | 56 |
| SP150020 | Duck intestine | CXM | — | 58 |
| SP150021 | Duck intestine | CXM, KF* | — | 64 |
| SP150027 | Duck intestine | CXM* | — | 74 |
| SP150036 | Duck intestine | CXM* | — | 94 |
| **SP150104** | Duck intestine | PRL, FEP, CXM, KF, CRO, ATM, K, S, NA, NOR, CIP, TE, F, C, SAM* | *bla*_SHV_, *bla*_TEM_, *bla*_CTX-M_ | 94,70 |
| T150072 | Healthy carrier | CXM* | *bla*_CTX-M_ | 65 |
| T150248 | Healthy carrier | SAM, CXM, K, NA, NOR, CIP, SXT, TE, C, AMC*, KF*, S*, F* | — | — |
| T150298 | Healthy carrier | S, CN, NA, NOR, TE, CXM*, F* | — | 192 |
| Ep271 | Diarrheal patient |  | *bla*_SHV_*, bla*_CTX-M_ | 100 |
| SP150175 | Duck intestine | SAM, K, CN, NA, NOR, CIP, SXT, TE, C, PRL*, AMC*, CXM*, KF*, S*, F* | *bla*_SHV_*, bla*_CTX-M_ | 262, 95, 77, 68 |
| SP150183 | Duck intestine | K, CN, NA, CXM*, KF*, F* | *bla*_SHV_*, bla*_CTX-M_ | 262, 95, 77, 68 |
| SP150185 | Duck intestine | PRL, S, TE, C | *bla*_SHV_*, bla*_CTX-M_ | 61 |
| SP150193 | Chicken intestine | PRL, K, S, CN, NA, NOR, CIP, SXT,C, SAM*, AMC*, CXM*,TE* | *bla*_SHV_*, bla*_CTX-M_ | 262, 95, 77, 68 |
| **SP150242** | Duck intestine | PRL, FEP, CXM, KF, CRO, ATM, S, NA, NOR,CIP, TE, F, C | *bla*_SHV_*, bla*_TEM_*, bla*_CTX-M_ | 92, 68 |
| SP150249 | Duck intestine | SAM, S, NA, SXT, TE, AMC*, CXM*, F* | *bla*_SHV_*, bla*_TEM_ | 83,36 |
| SP150253 | Duck intestine | CRO, S, NA, NOR, SXT, TE, SAM*, CXM*, CIP* | *bla*_TEM_ | 83,36 |
| SP150265 | Duck intestine | CXM, S* | — | 92 |
| SP150270 | Duck intestine | CXM*, KF* | *bla*_SHV_ | 121 |
| **SP140128** | Mutton | PRL, FEP, CXM, KF, CRO, ATM, K, S, NA, NOR, CIP, TE, C, SAM*, SXT*, F* | *bla*_CTX-M-55_*,* ***mcr-1*** | 113, 56 |
| **SP140150** | Chicken intestine | PRL, FEP, CXM, KF, CRO, ATM, K, S, NA, NOR, CIP, TE, F, C, SAM*, SXT* | *bla*_CTX-M-55_, ***mcr-1*** | 113, 56, 45 |
| SP140152 | Chicken intestine | PRL, CXM* | — | 76 |
| **SP140089** | Chicken meat | PRL, FEP, CXM, KF, CRO, ATM, K, S,NA, NOR, TE, F, C, SAM*, CIP*, SXT* | *bla*_CTX-M-55_, ***mcr-1*** | 113, 56, 45 |
| **SP140149** | Chicken intestine | PRL, FEP, CXM, KF, CRO, ATM, K, S, NA, NOR, CIP, TE, C, SAM*, LEV*, SXT*, F* | *bla*_CTX-M-55_, ***mcr-1*** | 113, 56, 45 |
| SP140148 | Chicken intestine | PRL, SAM, K, S, CN, NA, NOR, CIP, SXT, TE, C, AMC*, CXM*, F* | — | 199, 85, 72 |
| **SP140047** | Chicken intestine | PRL, FEP, CXM, KF, CRO, ATM, K, S, NA, NOR, TE, F, C, SAM*, CIP* | *bla*_SHV_, *bla*_CTX-M_ | 113 |
| SP140084 | Chicken meat | K, S, CN, NA, SXT, TE, PRL*, SAM*,CXM*, F* | *bla*_SHV_*, bla*_TEM_ | 85, 72 |
| SP140602 | Chicken intestine | PRL, SAM, S, CN, NA, SXT, TE, CXM* | *bla*_TEM_ | 169, 83, 73, 62, 38 |
| **SP140610** | Chicken intestine | PRL, SAM, FEP, CXM, KF, CRO, ATM, K, S, CN, NA, SXT, TE, C, F* | *bla*_SHV_, *bla*_TEM_, *bla*_CTX-M_ | 116, 84 |
| SP140618 | Chicken intestine | CXM, KF*, F* | — | 74 |
| SP140619 | Chicken intestine | CXM, KF*, F* | — | 93 |
| **SP140637** | Chicken intestine | PRL, FEP, CXM, KF, CRO, ATM, S, NA, SXT, TE, C, SAM* | *bla*_SHV_*, bla*_TEM_*, bla*_CTX-M_ | 95 |
| **SP140638** | Chicken intestine | PRL, SAM, CXM, KF, CRO, ATM, NA, SXT, TE, C, AMC*, F* | *bla*_TEM_, *bla*_CTX-M_ | 203, 61, 55 |
| SP140645 | Chicken intestine | S, SXT, TE, PRL*, SAM* | *bla*_TEM_ | 203, 64 |
| SP140674 | Chicken intestine | SAM, TE, C, AMC*, CXM* | *bla*_SHV_ | 203,64 |
| SP140692 | Duck meat | CXM*, KF*, F* | — | 66 |
| **SP140701** | Chicken meat | PRL, SAM, CXM, KF, CRO, ATM, K, S, NA, CIP, SXT, TE, C, FEP*, F* | *bla*_TEM_, *bla*_CTX-M_ | 203, 89, 72 |
| SP140724 | Chicken intestine | S, NA, TE, CXM*, KF*, F* | *bla*_SHV_ | 76,65 |
| SP140733 | Duck intestine | NA, SXT, TE, CXM*, CIP*, C* | *bla*_SHV_ | 93 |
| **SP140748** | Duck intestine | IMP, PRL, SAM, FEP, CXM, KF, CRO, ATM, S, CN, NOR, CIP, SXT, TE, F, C, MEM* | *bla*_SHV_, *bla*_TEM_, *bla*_CTX-M_ | 283, 80, 65 |
| **SP140749** | Duck intestine | PRL, SAM, FEP, CXM, KF, CRO, ATM, K, S, CN, NA, CIP, SXT, TE, F, C, NOR* | *bla*_TEM_, *bla*_CTX-M_ | 227, 95 |
| **SP140753** | Duck intestine | PRL, SAM, FEP, CXM, KF, CRO, ATM, K, S, CN, NA, NOR, CIP, SXT, TE, F, C | *bla*_SHV_, *bla*_TEM_, *bla*_CTX-M_ | 227, 95, 72 |
| **SP140754** | Duck intestine | PRL, SAM, FEP, CXM, KF, CRO, ATM, K, S, CN, NA, NOR, CIP, SXT, TE, F, C | *bla*_TEM_, *bla*_CTX-M_ | 227, 95, 72 |
| SP140771 | Chicken intestine | CXM, KF*, S*, TE*, F* | *bla*_SHV_ | 124 |
| SP140791 | Pork | NA, TE | — | 172 |
| SP140807 | Chicken intestine | PRL, S, SXT, TE, SAM* | *bla*_TEM_ | 213, 67 |
| SP140813 | Chicken intestine | S, NA, SXT, TE, SAM*, F* | *bla*_TEM_ | 99, 87, 64 |
| SP140837 | Duck intestine | CXM | *—* | 79 |
| SP140839 | Duck meat | PRL, CXM* | *—* | 79 |

Antibiotics abbreviations are as follows: PRL, piperacillin; SAM, ampicillin/sulbactam; FEP, cefepime; CXM, cefuroxime; KF, cephalothin; CRO, ceftriaxone; ATM, aztreonam; K, kanamycin; S, streptomycin; CN, gentamicin; NA, nalidixic acid; NOR, norfloxacin; CIP, ciprofloxacin; SXT, trimethoprim/sulfamethoxazole; TE, tetracycline; F, furadantin; C, chloramphenicol.

^a^: ESBLs-producing strains were in bold.

*:Intermediate sensitivity to antibiotics.

—: All the genes tested were negative.
